# The comparison of plasma fibronectin in term and preterm delivery: A cross-sectional, descriptive-analytical study

**DOI:** 10.18502/ijrm.v18i1.6191

**Published:** 2020-01-27

**Authors:** Zahra Moradi, Parvin Moradi, Mohamad Hassan Meshkibaf, Mehrnoosh Aleosfoor, Mehdi Sharafi, Saeedeh Jafarzadeh

**Affiliations:** ^1^School of Nursing, Fasa University of Medical Sciences, Fasa, Iran.; ^2^Department of Obstetrics and Gynecology, Medical School, Fasa University of Medical Sciences, Fasa, Iran.; ^3^Department of Clinical Biochemistry, Fasa University of Medical Sciences, Fasa, Iran.; ^4^Non-Communicable Diseases Research Center, Fasa University of Medical Sciences, Fasa, Iran.

**Keywords:** Premature birth, Fibronectin, Maternal serum screening tests.

## Abstract

**Background:**

Preterm delivery is one of the main causes of infant death. Therefore, prediction of preterm delivery may eliminate a large number of prenatal complications.

**Objective:**

The present study aimed to understand if preterm delivery can be predicted by assessing maternal plasma fibronectin concentration.

**Materials and Methods:**

Serum samples from 105 pregnant women participating in this study were collected. The plasma fibronectin were measured at 24-28 wk of gestation and again at 32-36 wk of gestation. Unfortunately, only 65 of the 105 pregnant women, returned for the second sampling. The plasma fibronectin was analyzed using ELISA method and its concentration in term and preterm deliveries was compared. The delivery dates of all the women were also recorded.

**Results:**

Out of 105 pregnant women, 28 delivered preterm (26.7%). The Plasma fibronectin concentrations in women with preterm delivery were higher than in those who delivered at term (p = 0.001). Accordingly, Plasma fibronectin concentrations were significantly higher in the second serum samples (p = 0.01). Plasma fibronectin concentrations was also higher in obese women and in those suffering from preeclampsia (p = 0.12) and gestational diabetes (p = 0.81).

**Conclusion:**

Plasma fibronectin concentrations test could be used as an optional screening test for preterm delivery at 28 to 34 wk of gestation in pregnant women who prefer to avoid vaginal sampling.

## 1. Introduction 

Preterm delivery is defined as delivery before 37 wk of gestation or 259 days after the last menstrual period (LMP) (1, 2). It is one of the main reasons for infant mortality in the world, accounting for 35% of infant deaths annually (3, 4). Preterm delivery is still responsible for 70% of neurological complications, disabilities, and deaths, which impose a substantial economic healthcare burden (5, 6). Several methods are available for diagnosing, including investigation of cervical dilation, assessment of cervical activity with a dynamometer, performing sonography to determine gestational age, evaluation of cervical length using abdominal or vaginal ultrasound, and evaluation of vaginal fibronectin levels and salivary estriol (2). However, identification by a reliable screening marker can predict preterm delivery before the incidence of clinical symptoms. Vaginal fibronectin concentration is a reliable marker in the prognosis of preterm delivery. However, its effectiveness may be limited because of anxiety and cultural misgivings among pregnant women about the method of sampling, fibronectin secretion increases due to endothelial stress (7-10). Thus, in the case of preterm delivery, preeclampsia, and intrauterine growth restriction (IUGR), fibronectin concentration increases weeks and even months before delivery. It is rarely found in discharges after the 21${}^{\mathrm{st}}$ wk of gestation. However, it increases again before delivery. Thus, the early presence of fibronectin in cervical and vaginal discharges can predict preterm delivery (11, 12).

For fetal fibronectin test, most samples are taken through the vaginal route when the process of preterm delivery begins. Because fibronectin concentration increases earlier in maternal plasma compared with vaginal discharges, Zygmunt proposed that high Plasma fibronectin levels could be effective in the prognosis of preterm delivery. Plasma fibronectin concentration follows an ascending trend in high-risk pregnancies (13). Assessment of Plasma fibronectin concentration is a simple, non-invasive, and accurate method for investigation of endothelial function (11). In Iran, pregnant women are reluctant to undergo vaginal examination during pregnancy, believing it is hazardous to infant health and may itself cause preterm delivery. The availability of another sampling option may help the patient as well as the physician to evaluate the chance of preterm delivery. Because blood sampling is simpler and less invasive than vaginal sampling, the purpose of the present study is to evaluate and compare Plasma fibronectin concentrations in women that delivered preterm and at term?”

## 2. Materials and Methods

This cross-sectional, descriptive-analytical study was conducted in 105 pregnant women after considering the inclusion and exclusion criteria. The pregnant women had been referred to the gynecology clinic in Vali-e-Asr Hospital, Fasa, Iran for routine prenatal care between March 2014 and April 2016.

The inclusion criteria included age (18-35 years old), single pregnancy, gestational age of 24-28 and 32-36 wk; the exclusion criteria included a history of receiving tocolytic agents (terbutaline, ritodrine, magnesium sulfate, salbutamol, and isoxsuprine) and suffering from chronic hypertension, diabetes, renal problems, and inflammatory disorders such as lupus. The gestational age was determined according to LMP. In case of doubt about the date of LMP, the first-trimester sonography was taken into consideration. Demographic data (parents' ages, occupations, and education levels) and obstetric information were collected for each participant. A venous blood sample was collected, serum separated, and kept at μ70°C. All the samples were assessed for fibronectin concentrations using 96-well enzyme-linked immunosorbent assay (ELISA) kits. The first sampling was undertaken at 24-28 wk of gestation and second sampling at 32-36 wk of gestation. However, samples could only be taken from 65 women during the second sampling period. All participants were followed until delivery and information about infants' weight, type of delivery, and gestational age at the time of delivery was collected and recorded. Plasma fibronectin concentrations were assessed using ELISA kits (BE59341 IBL international GMBH).

### Ethical considerations

Women were informed about the study and those who were interested to join, provided written informed consents. This article was extracted from a research proposal (93104) approved by Fasa University of Medical Sciences.

### Statistical analysis

All data were analyzed using SPSS statistical software (IBM Corp. Released 2013. IBM SPSS Statistics for Windows, Version 22.0. Armonk, NY: IBM Corp.”). Descriptive statistics such as mean, standard deviation (SD), and percentage were used. The chi-square independent and paired t-tests and a logistic regression analysis were also employed. In addition, the receiver operating characteristic (ROC) curve was used to assess the cutoff points. The significance level was set at α< 0.05.

## 3. Results

The results showed no significant differences between the women with term and preterm deliveries with respect to the mother's age, father's age, and the interval between marriage and first pregnancy (p = 0.51). There were also no significant differences between the women with term and preterm deliveries with respect to occupation, education level, consanguineous (family) marriage, parity, history of miscarriage, husband's cigarette smoking status, family history of preterm delivery, contraception method, and utilization of assisted reproductive techniques (ART) (p = 0.49). However, the body mass index (BMI) before pregnancy was higher among women in the preterm delivery group (p = 0.01), which continued throughout pregnancy. Higher fibronectin concentration was accompanied by lower infant weight (p = 0.001) (Table I).

The results revealed that 28 participants had preterm delivery (26.7%). Among the women with preterm delivery, 13 had natural vaginal delivery (NVD) (46.4%) and 15 had a cesarean section(C/S) (53.6%). These measures (NVD or C/S) were 57.1% and 42.9%, respectively, in the term delivery group (p = 0.33). According to the results, two women in the preterm delivery group (7.1%) and three women in the term delivery group (3.9%) had unplanned pregnancies (p = 0.009). Besides, 53.6% of the women in the preterm delivery group and 31.2% of those in the term delivery group had a family history of diabetes and hypertension (p = 0.03). Additionally, 17.9% of the women in the preterm delivery group and 10.4% of those in the term delivery group reported a history of preterm delivery in their mothers or sisters. However, the difference was not statistically significant (p = 0.30) (Table I). Out of 65 women who took part in the second sampling, 11 had preterm delivery and 54 had term delivery. At the first sampling (24-28 wk), the results of fibronectin concentrations revealed no significant difference (p = 0.66). Plasma fibronectin concentrations in the term delivery group decreased in the second sampling, but the difference was not statistically significant (p = 0.16). However, in contrast, the mean plasma fibronectin concentration significantly increased in the preterm delivery group (p = 0.01). In other words, the increase in gestational age was accompanied by a significant increase in the fibronectin concentration in the preterm delivery group (p = 0.001) (Table II and III). With respect to pregnancy complications, the incidence of diabetes and hypertension was higher in women in the preterm delivery group. Moreover, plasma fibronectin concentrations were significantly higher in the women suffering from gestational diabetes and preeclampsia (p = 0.009) (Tables IV and V).

**Figure 1:**
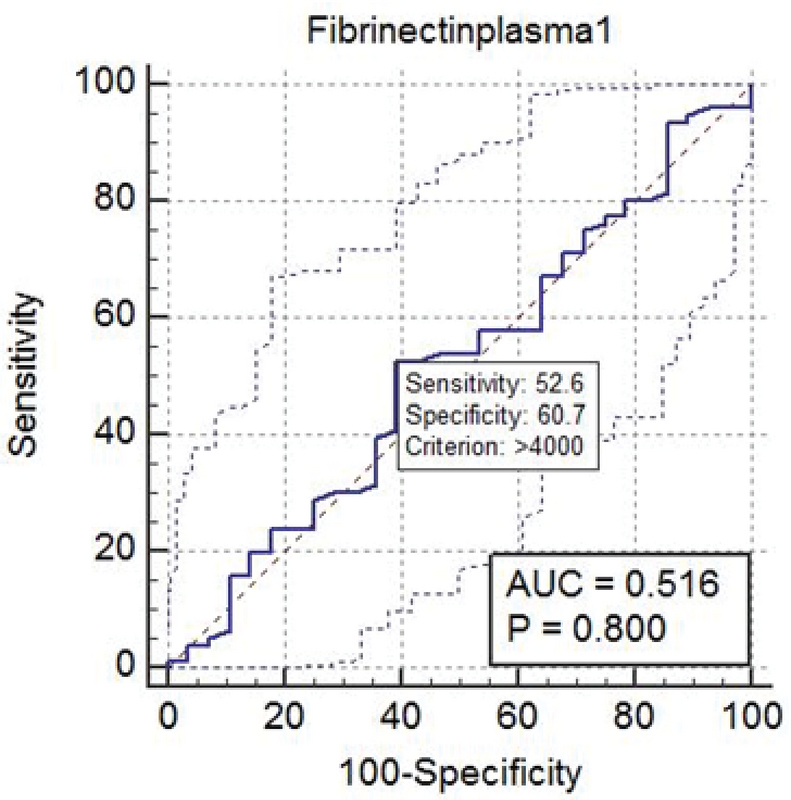
Receiver operating characteristic curve for fibronectin plasma 1.

**Figure 2:**
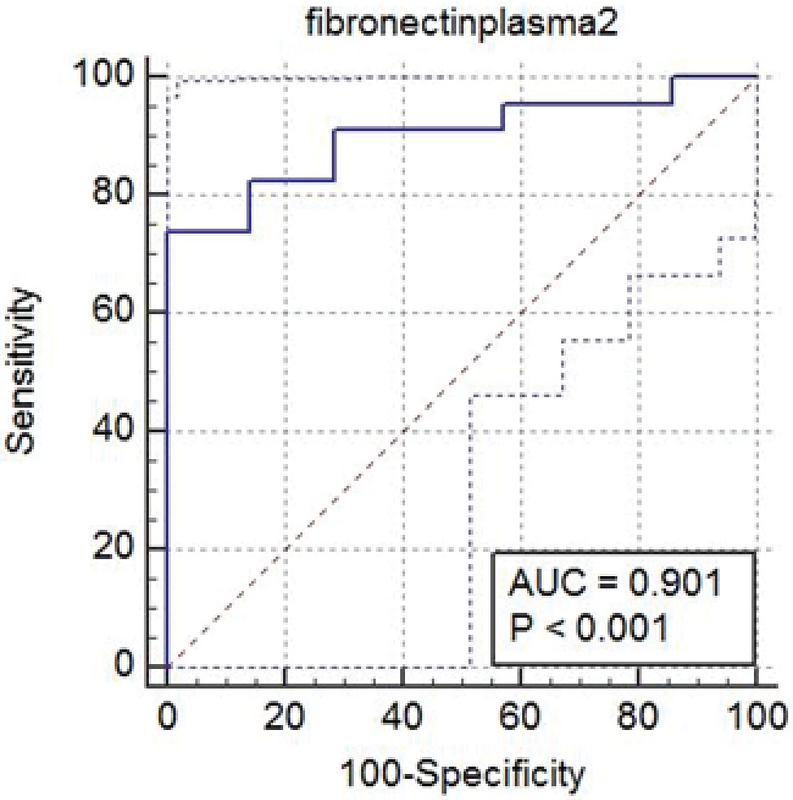
Receiver operating characteristic curve for fibronectin plasma 2.

According to the ROC curve, the best cutoff point in the first sampling was 1.750 ng/ml with a sensitivity of 80.26%, specificity of 17.85%, positive predictive value of 73/08%, and negative predictive value of 26/92%. In the second sampling, the best cutoff point was 4,226 ng/ml with a sensitivity of 100%, specificity of 74%, positive predictive value of 76/67%, and negative predictive value of 23/33%. According to these measures, this test should be used after the 28${}^{\mathrm{th}}$ wk of gestation (Figures 1, 2).

**Table 1:** Frequency distribution and comparison of demographic, labor, and individual factors in two groups


**Personal and delivery details, and maternal factors**		**Preterm N = 28 (26.7%)**	**Term N = 77 (73.3%)**	**Total N = 150 (100%)**	**P-value**
Mother's age (yr) *		29.47 ± 5.56	28.57 ± 6.50	28.81 ± 6.257	0.51*
Husband's age (yr)*		35.39 ± 7.28	33.45 ± 6.03	33.97 ± 6.417	0.17
The average age of marriage for women (yr) *		22.21 ± 5.39	21.70 ± 4.66	21.84 ± 4.84	0.63
Time span between the age of marriage and the first pregnancy (yr) *		2.25 ± 1.79	2.57 ± 3.20	2.49 ± 2.84	0.63
Gestational age in the first sampling (wk) *		26.45 ± 2.68	26.16 ± 2.33	26.1 ± 2.98	0.87
Gestational age in the second sampling (wk) *		31.18 ± 3.10	32.33 ± 2.74	30.90 ± 2.35	0.42
Gestational age at birth (wk) *		35.12 ± 1.10	39.25 ± 2.13	38.24 ± 2.03	0.012
Mother's weight at first sampling (kg) *		65.3 ± 10.56	60.42 ± 10.74	61.72 ± 10.867	0.42
Maternal weight at delivery (Kg) *		74.25 ± 12.33	67.54 ± 12.24	69.33 ± 12.566	0.17
Maternal BMI before pregnancy *		26.24 ± 346	24.18 ± 3.88	24.73 ± 3.87	0.01
Delivery type **					
	NVD	13 (46.4)	44 (57.1)	
	C/S	15 (53.6)	37 (42.9)	0.33**
Mother's job **					
	Housewife	22 (78.6)	71 (92.2)	
	Employed	6 (21.4)	6 (7.8)	0.15
Husband's job**					
	The manual worker	2 (7.1)	16 (20.8)	
	Employee	8 (28.6)	16 (20.8)	
	Freelancer	18 (64.3)	45 (58.4)	0.34
Mother's education **					
	Illiterate	2 (7.1)	1 (1.3)		
	Under the diploma	7 (15)	27 (35.1)	
	Academic	19 (67.8)	49 (63.7)	0.37
Husband's education **					
	Illiterate	1 (3.6)	5 (6.5)		
	Under the diploma	15 (53.6)	53 (68.8)	
	Academic	12 (42.8)	19 (24.7)	0.08
Gravid **					
	1	13 (6.4)	27 (35.1)		
	2	8 (28.6)	27 (35.1)	
	3≥	7 (25)	23 (29.8)	0.70
Abortion history **					
	Yes	4 (14.3)	19 (24.7)		
	No	24 (85.7)	58 (75.3)	0.25
Smoking by husband **					
	Yes	6 (21.4)	14 (18.2)	
	No	22 (78.6)	63 (81.8)	0.70
Preterm labor history **					
	Yes	23 (82.1)	69 (89.6)	
	No	5 (17.9)	8 (10.4)	0.30
Use of fertility assisted methods **					
	Yes	2 (7.1)	3 (3.9)	
	No	26 (92.9)	74 (96.1)	0.49
Disease in the current pregnancy **					
	GDM	4 (14.28)	1 (1.31)	
	PEC	4 (14.28)	2 (2.63)	
	Infection	7 (25)	19 (25)	
	Vaginal bleeding	1 (3.57)	10 (13.15)	0.009
* Mean ± SD, **n (%), P-value*, Qualitative variables were analyzed using an independent t-test, P-value**, Chi-square test NVD: Normal vaginal delivery, C/S: Cesarean section, GDM: Gestational diabetic mellitus, PEC: Preeclampsia					

**Table 2:** Comparison of mean plasma concentrations of fibronectin in two groups in double sampling


**Variable**	**Fibronectin plasma1**	**Fibronectin plasma2**	P-value*
Term	7727.50 ± 11506.734	4059.57 ± 3175.744	0.16
Preterm	6726.43 ± 7174.916	10507.14 ± 5477.106	0.01
P-value* *	0.668	0.001	
* Mean ± SD, Qualitative variables were analyzed using an independent and dependent t-test and quantitative variables were analyzed using logistic regression test, P-value*, paired sample t-test; P-value**, independent t-test			

**Table 3:** Comparison of mean of fibronectin concentrations in the first and second sampling groups


**Variable**	**N**	**Mean ± SD**	**p-value**
Fibronectin plasma1	30	4366.66 ± 5209.09	
Fibronectin plasma2	30	5564 ± 4642.26	0.009
* Mean ± SD, P-value, paired sample t-test			

**Table 4:** Comparison of fibronectin concentration in women with gestational diabetes and non-diabetes individuals


**Fibronectin GDM**	**Fibronectin plasma 1**		**Fibronectin plasma 2**	
	Mean ± SD	N	Mean ± SD	N
Yes	6508.75 ± 5175.02	8	6166.67 ± 3663.78	3
No	7537.08 ± 10831.50	96	5497.04 ± 4791.46	27
P-value	0.791		0.817	
* Mean ± SD, P-value: Independent t-test, GDM: Gestational diabetic mellitus				

**Table 5:** Comparison of fibronectin concentration in women with preeclampsia and non-affected patients


**Fibronectin Preeclampsia**	**Fibronectin plasma 1**		**Fibronectin plasma 2**	
	Mean ± SD	N	Mean ± SD	N
Yes	9581.67 ± 7356.95	6	12600	1
No	7327.96 ± 10669.99	96	5321.38 ± 4526.73	29
P-value	0.612		0.125	
* Mean ± SD, P-value: Independent t-test				

## 4. Discussion

The results indicated that plasma fibronectin concentration was higher in the women who had given birth to their children before 37 wk of gestation, which is consistent with the study by Zigmunt (13). Forouhari and colleagues compared maternal plasma fibronectin concentration in three study groups, including women with symptoms and risk factors of preterm delivery, women with symptoms but without risk factors of preterm delivery, and healthy pregnant women. The results showed that plasma fibronectin concentration was significantly higher in the preterm delivery group compared with the term delivery group (2). Severens-Rijvers also evaluated fibronectin concentration before pregnancy and at 12 and 16 wk of gestation. Based on the results, fibronectin concentration was higher in women with maternal placental syndrome and had increased since before pregnancy up to the 16${}^{\mathrm{th}}$ wk of gestation. However, in women without the maternal placental syndrome, this value increased up to the 12${}^{\mathrm{th}}$ wk of gestation but decreased below the pre-pregnancy value at wk 16^th^ (11). The results of our study are consistent with the findings of the above studies. Thus, it can be concluded that plasma fibronectin concentration follows an ascending trend in high-risk pregnancies. It usually increases weeks and even months before the incidence of complications, such as preterm delivery, preeclampsia, and IUGR (13). Increased plasma fibronectin concentration may result from its secretion from damaged endothelial cells occurring in placentas of women experiencing preterm delivery (13). In this regard, vascular problems and inflammatory markers affect plasma fibronectin concentration. These changes are similar to those occurring in IUGR and preeclampsia (14). In the present study, only 65 out of the 105 participants took part in the second sampling. Among these women, 58 and 7 belonged to the term and preterm delivery groups, respectively. Comparing the results of fibronectin concentration in the first and second sampling for those who attended for the second time showed that an increase in gestational age was accompanied by an increase in fibronectin concentration, which is consistent with studies by Ekaidem and Dane (14, 15). Production and secretion of fibronectin into body fluids is accompanied by the natural growth of placental and trophoblastic villi, which increase with an increase in gestational age (14). The results of the present study revealed that an increase in BMI before pregnancy and an increase in the mother's weight at the time of delivery led to an increase in plasma fibronectin concentration. In contrast, an early increase in plasma fibronectin concentration leads to preterm delivery. Moreover, five women developed gestational diabetes and six developed preeclampsia. Of these, eight in total underwent preterm delivery. Fibronectin concentration was also higher in women with gestational diabetes and hypertension. These results were similar to those obtained by Ekaidem and Rasanen, which showed that plasma fibronectin concentration and the risk of preeclampsia were higher among obese women (14, 16). The relationship between increased plasma fibronectin concentration and risk of preeclampsia in obese women may be attributed to obesity complications. Hypertension in obese individuals can reduce placental perfusion and increase vascular problems, which eventually increase the production and secretion of fibronectin as well as the production of pro- inflammatory products (14, 16). Parker and Kim also demonstrated that maternal obesity was accompanied by increased risk of preterm delivery due to medical complications such as gestational diabetes and blood pressure disorders. Thus, interventional strategies for reducing the risk of gestational diabetes and blood pressure disorders could decrease the risk of preterm delivery as well (17, 18). Thus, evaluation of mothers' weight and BMI before pregnancy and at the first visit after that, nutritional consultation, and provision of necessary care services could play an important role in maternal and infantile health and prevention of undesirable complications. Health behaviors and lifestyle modifications, as well as weight adjustment, are essential during pregnancy in order to have a healthy delivery and avoid complications and negative outcomes (19-22). The results of the present study showed that plasma fibronectin concentration decreased in the second sampling compared with the first sampling in the term delivery group. Considering the fact that the increase in plasma fibronectin concentration is indicative of preterm delivery, this finding followed the normal trend of changes in fibronectin concentration during pregnancy (10). However, this measure was significantly higher in the second sampling compared with the first sampling in the preterm delivery group. This implies that factors leading to preterm delivery follow an ascending trend and that plasma fibronectin concentration increases over time.

In the studies by Zigmunt and Forouhari (2, 13), plasma fibronectin concentration was assessed in women who showed symptoms of preterm delivery. However, the present study aimed to determine whether plasma fibronectin concentration could be used as a screening test. Therefore, the participants were selected from women who had no symptoms of preterm delivery. In addition, blood was taken at two stages with a 4-6 wk interval in order to determine the best time for performing the test. Zigmunt *et al*. also reported the highest plasma fibronectin concentration (600 ± 160) in the women who had given birth to their children before 32 wk of gestation (21). In Forouhari's study, plasma fibronectin concentration was higher in the preterm delivery group compared with the term delivery group, with the best cutoff point being 700 ng/ml. The difference between the results may be because of the kits utilized. Moreover, Forouhari reported sensitivity, specificity, positive predictive value, and negative predictive value to be 100%, 61.1%, 54.3%, and 100%, respectively. The present study benefitted from higher specificity (2). However, Zigmunt *et al*. measured fibronectin concentration using the nephelometry method and reported the cutoff point to be 450 ng/ml. Indeed, sensitivity, specificity, positive predictive value, and negative predictive value were reported to be 50%, 95%, 50%, and 90%, respectively (13). As mentioned above, Zigmunt assessed fibronectin concentration using the nephelometry method, while ELISA is a more accurate technique. In the present study, the prevalence of preterm delivery was 26.7%. Various prevalence rates have been reported in different studies. For instance, Forouhari *et al*. reported the prevalence of preterm delivery to be 31.6% in Shiraz (2). Additionally, Shojae and Shariati showed that the prevalence of the condition was 7.2% in Bojnourd and 15.4% in Tehran and around the world (6, 23). These differences may be attributed to various causes of preterm delivery in different cultural and geographical regions. The findings of the present study demonstrated that an increase in plasma fibronectin concentration resulted in a decrease in birth weight. This decrease may have resulted because of preterm delivery. Similarly, Forouhari and Zigmunt found that an increase in plasma fibronectin concentration caused a decrease in birth weight (2, 13). Given that only two studies have been conducted on the assessment of plasma fibronectin concentration for predicting preterm delivery, limited studies were available for comparison of the results. Moreover, sampling was not undertaken in several stages in the previous studies to evaluate changes in fibronectin concentration in preterm delivery. Thus, further studies may help to achieve more reliable results regarding this issue.

## 5. Conclusion

Plasma fibronectin concentration can predict the process of preterm delivery earlier than the fetal fibronectin test. Therefore, it can be used as a screening test in the prognosis of preterm delivery. We suggest the best time for analysis to be after 30 wk of pregnancy. We have undertaken another study to evaluate and compare fetal and plasma fibronectin concentrations at the same time in the same patients.

##  Conflicts of Interest

The authors declare no conflicts of interest.
